# Predicting demand for long-term care using Japanese healthcare insurance claims data

**DOI:** 10.1265/ehpm.22-00084

**Published:** 2022-10-29

**Authors:** Jumpei Sato, Naohiro Mitsutake, Masaru Kitsuregawa, Tomoki Ishikawa, Kazuo Goda

**Affiliations:** 1 Institute of Industrial Science, The University of Tokyo, Meguro-ku, Tokyo, Japan; 2 Institute for Health Economics and Policy, Minato-ku, Tokyo, Japan

**Keywords:** Long-term care service, Healthcare insurance claims database, Demand prediction

## Abstract

**Background:**

Driven by the rapid aging of the population, Japan introduced public long-term care insurance to reinforce healthcare services for the elderly in 2000. Precisely predicting future demand for long-term care services helps authorities to plan and manage their healthcare resources and citizens to prevent their health status deterioration.

**Methods:**

This paper presents our novel study for developing an effective model to predict individual-level future long-term care demand using previous healthcare insurance claims data. We designed two discriminative models and subsequently trained and validated the models using three learning algorithms with medical and long-term care insurance claims and enrollment records, which were provided by 170 regional public insurers in Gifu, Japan.

**Results:**

The prediction model based on multiclass classification and gradient-boosting decision tree achieved practically high accuracy (weighted average of Precision, 0.872; Recall, 0.878; and F-measure, 0.873) for up to 12 months after the previous claims. The top important feature variables were indicators of current health status (e.g., current eligibility levels and age), risk factors to worsen future healthcare status (e.g., dementia), and preventive care services for improving future healthcare status (e.g., training and rehabilitation).

**Conclusions:**

The intensive validation tests have indicated that the developed prediction method holds high robustness, even though it yields relatively lower accuracy for specific patient groups with health conditions that are hard to distinguish.

## Graphical Abstract

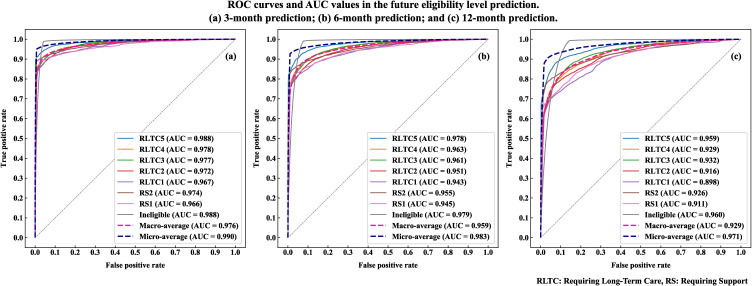

## Background

Quantitative estimation of future service demand is crucial to sustain and advance a sophisticated healthcare system. Several previous studies have aimed to predict future service demand [1–11]. This paper presents our study for exploring an effective model to predict individual-level future long-term care demand using previous insurance claims data from medical services and long-term care services in Japan.

Japan is experiencing a super-aging society previously unwitnessed by any country in the world. A universal public medical insurance system was established in Japan in 1961; however, it merely covers the treatment of injuries and diseases for citizens [12, 13]. In 2000, the Japanese government introduced a public long-term care insurance system to extend healthcare support for the elderly [14, 15]. This new insurance program covers daily life assistance/support (e.g., eating and bathing assistance and housework support), rehabilitation at home, and visiting nursing care, none of which are provided by the conventional medical insurance [14, 16]. The long-term care insurance effort has grown steadily since its launch. Currently, medical and long-term care insurance are the two major backbones of Japan’s healthcare system. In 2002, the national long-term care expenditure was 5.2 trillion JPY (approximately 520 billion USD) [17], which accounted for only 6.2% of the total social security expenditure (83.6 trillion JPY, equivalent to around 8,360 billion USD) [18]. In 2018, the long-term care expenditure reached 10.7 trillion JPY (around 1,070 billion USD), which accounted for 8.8% of the total social security expenditure [19]. The Japanese government plans to further extend the long-term care insurance to significantly enhance the preventive care and suppress the growth of medical expenditures. Macroscopic analysis revealed that the long-term care expenditure would reach up to 25.8 trillion JPY (2,580 billion USD) by 2040 [19].

Japan’s public long-term care insurance categorizes all individuals aged ≥65 years into eight eligibility levels according to their requirement for long-term care (Table 1) [14]. Individuals are provided with long-term care services according to their approved eligibility levels. Requiring Long-Term Care 5 (RLTC5) includes individuals with the most severe health issues and intensive care demands, whereas Requiring Support 1 (RS1) includes individuals with minor healthcare problems. Sufficiently healthy individuals are ineligible and do not receive care. Other countries, such as France and Germany, have employed similar frameworks [20–22].

**Table 1:** Eligibility levels for long-term care insurance in Japan.

**Eligibility ** **level**	**Health status**	**Examples of long-term care services**	**Service provision allowance**
RLCT5	Requiring 24-h thorough care (e.g., confined to bed)	Eating and bathing assistance (daily), visiting nursing care (twice a week)	360,650 JPY per person-month
RLCT4	Requiring 24-h specific care (e.g., requiring a wheelchair)	Eating and bathing assistance (4 times a week), visiting nursing care (weekly)	308,060 JPY per person-month
RLCT3	Requiring assistance for daily life (e.g., requiring a walker)	Housework support (3 times a week), visiting nursing care (weekly)	269,310 JPY per person-month
RLCT2	Requiring partial assistance for daily life (dementia of mild degree)	Housework support (weekly), visiting nursing care (every 2 weeks)	196,160 JPY per person-month
RLCT1	Requiring partial assistance for specific motion (e.g., difficulty with excretion)	Housework support (weekly), visiting rehabilitation (weekly)	166,920 JPY per person-month
RS2	Requiring preventive assistance (e.g., unable to washing their back)	Preventive training (twice a week), housework support (every 2 weeks)	104,730 JPY per person-month
RS1	Possibly requiring preventive assistance (e.g., unable to clean a room)	Preventive training (once a week), housework support (every 2 weeks)	50,030 JPY per person-month
Ineligible	None of the above (healthy)	No services to be provided	–

Certification of eligibility is provided by the local government authority on demand. An assigned official consults the elderly individual or their family to inspect their physical and psychological health status and cognitive ability in accordance with the eligibility criteria prescribed by the government. The official then decides their eligibility and designs a long-term care plan. Currently, the local government can only roughly predict future long-term care demand using population-based estimation techniques. Individual-level prediction based on medical and long-term care history is challenging but potentially improves the accuracy.

Accurate prediction of future long-term care demand helps regional authorities to manage and plan their long-term care resources. Long-term care involves a package of various services that require human resources with different skills and licenses. For example, visiting nursing care is only conducted by licensed nurses by law. Therefore, enabling authorities to precisely estimate future demand (even several months after) of specific services in an area of charge would allow them to act preemptively to secure the necessary workforce and would likely improve the stability and efficiency of regional long-term care resources.

In addition, individual-level prediction can help to prevent the escalation of regional long-term care demand. Previous studies reported that preventive care, such as rehabilitation and exercise, could improve physical and cognitive function and thus prevent the deterioration of health status and the increase in individuals’ eligibility levels [23–26]. Early provision of preventive care based on individual-level prediction will help to maintain individuals’ health status and suppress the growth of regional long-term care demand.

The present study aimed to accurately predict individual-level future long-term care demand using information about medical services and long-term care services. Medical and long-term care services are separately managed in Japan’s healthcare system; however, their effects on each other are reciprocal. For example, onset of stroke or dementia are primary risk factors that worsen an individual’s future healthcare status and increase the demand for long-term care services [26, 27]. In contrast, a continuous rehabilitation service will improve the physical condition to help the recovery of individuals’ daily living capabilities [28, 29]. Similarly, bathing assistance services may improve individuals’ sanitary, psychological and physical conditions and suppress pathological aggravation.

This paper presents an effective method to predict individual-level future long-term care demand using previous insurance claims data of medical and long-term care services. We designed two different discriminative models and trained them using three different learning algorithms with medical and long-term care insurance claims provided by 170 regional public insurers covering 42 local government areas in Gifu, Japan. Because different insurers separately manage medical insurance claims and long-term care insurance claims, this study utilized insurance enrollment history records that allow us to combine and integrate all the claims for each individual. Intensive comparative validation tests were performed to confirm the practical effectiveness. To our knowledge, this is the first study to use data from previous healthcare insurance claims to predict future long-term care demand. A preliminary version of this work has been reported elsewhere [30].

## Methods

We developed an effective prediction method to predict future long-term care demand using previous insurance claims data of medical and long-term care services at the individual level. We aimed to predict individuals’ long-term care eligibility levels at 3, 6, and 12 months using their insurance claims of medical services, and, if available, their long-term care services for the past 12 months.

**Fig. 1:**
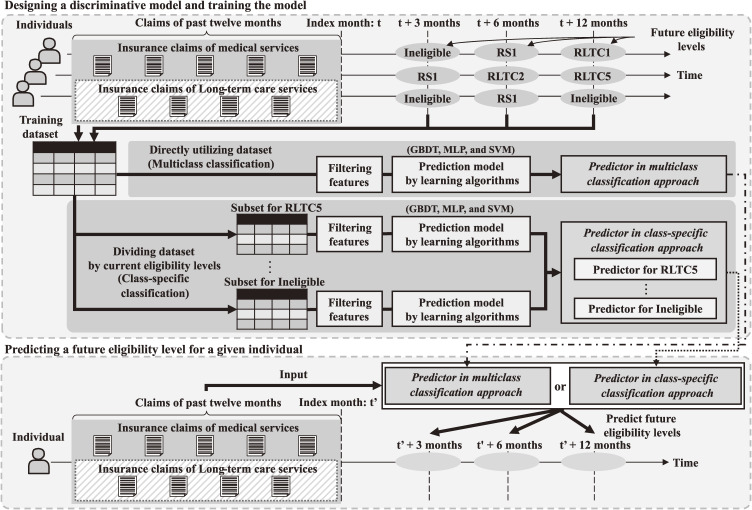
Study framework for future prediction of individuals’ long-term care eligibility levels.

We employed a supervised machine learning approach. Figure 1 outlines the study framework to develop the prediction method. First, discriminative models were designed to input previous insurance claims and output a future eligibility level for each individual. Second, the designed models were trained using state-of-the-art learning algorithms with training datasets. Third, the trained models were applied to different test datasets to evaluate their effectiveness.

### Model design

Two different modeling approaches were employed in the design process of the discriminative models. The first approach was multiclass classification, in which all training datasets were directly employed to build a single predictor. Although multiclass classification was widely utilized, previous machine learning studies indicated that imbalanced learning samples resulted in low prediction accuracy [31–33]. The distribution of long-term care eligibility levels showed a significant imbalance. In 2018, a statistical report [34] revealed that only 1.5% of the target population were assigned to RLTC5, whereas 81.6% were ineligible. Individuals in different eligibility levels were assumed to have drastically different health conditions. It may not be possible for a single prediction mechanism to cover all eight eligibility levels with reasonably high accuracy. Therefore, in addition to the multiclass classification approach, we also explored the class-specific classification approach [35, 36], in which the training dataset was divided into disjointed subsets based on individuals’ current eligibility levels, and a separate predictor was built for each subset.

The specific model design process can be divided into two steps as follows.

Step 1: For the training dataset, we obtained medical and long-term care insurance claims from the past 12 months and future long-term care eligibility levels of 3, 6, and 12 subsequent months from the database, transformed them into a longitudinal form, and generated a feature variable vector for each individual. The feature variables included (1) the frequency of occurrence of each code for disease diagnosis, medical treatment, drug prescription, medical instrument, and long-term care service; (2) the medical expenditure of each month; (3) the number of claims in the last 1, 3, 6, and 12 months; (4) sex and age; (5) current eligibility levels; and (6) future long-term care eligibility levels for the following 3, 6, and 12 months.

Step 2: In the multiclass classification approach, we applied a filtering technique using the F-value of the analysis of variance (ANOVA) to select 150 statistically representative codes [37–39]. In the class-specific classification approach, we performed the same step separately for each subset. This filtering was crucial, as the feature variable vector space was likely to be sparse. For example, disease diagnosis codes had more than 1,000 for cardinality. The use of large numbers of features has previously been shown to induce overfitting of the model [38]. Furthermore, the naive calculation consumed a lot of memory and hindered the learning process. Modern machine learning algorithms have penalty terms that mitigate multicollinearity problems. Multicollinearity was previously reported to have little effect on the classification accuracy of the tree-based algorithm [40]; thus, the present study only used ANOVA-based filtering.

### Model training

We employed three major learning algorithms, namely, gradient-boosting decision tree (GBDT) [41], multilayer perceptron (MLP) [42], and support vector machine (SVM) [43], to train the designed models using training datasets. The hyperparameters were tuned for each learning algorithm based on the grid-search method, and threefold cross-validation was performed. As per the model design process, the training datasets were used directly in the prediction models designed in the multiclass classification approach. In contrast, in the class-specific classification approach, the training dataset was divided, and each subset was used in the designed prediction models. Notably, we applied the downsampling method for the training dataset to mitigate the imbalance effect [31–33].

### Model test

We applied the models to test datasets and clarified the prediction accuracy to evaluate the effectiveness of the designed and trained models. Precision, Recall, and F-measure (the harmonic mean of Precision and Recall) were used as evaluation indicators for the prediction of each eligibility group. In addition, (unweighted) averages and weighted averages of Precision, Recall, and F-measure over all the eligibility levels were obtained as overall evaluation indicators, in which the weighted average was calculated by (1) summing the indicator value multiplied by the number of individuals for each eligibility level and (2) dividing the total sum by the total number of individuals [44]. We conducted an intensive study to compare the two modeling approaches and three learning algorithms and verified the robustness of the accuracy.

### Dataset

We used anonymized medical and long-term care insurance claims provided by 170 regional public insurers covering 42 local government areas in Gifu, Japan. The dataset included 49,991,060 medical insurance claims and 5,754,329 long-term insurance claims, which were recorded from April 2015 to March 2018. We also used anonymized insurance enrollment history information (2,934,609 records) to combine medical and long-term care insurance claims for each individual. The anonymization had been performed before being provided to us; personal identifiers (e.g., names and insured numbers) were removed or irreversibly converted and instead encoded identifiers (hash values generated from them) were appended. The dataset statistics are summarized in Table 2. The eligibility levels (RLTC5 to RS1) were explicitly described in long-term care insurance claims. Individuals who were recorded in the enrollment history but not provided with any long-term care were deemed to be ineligible.

**Table 2:** Dataset used in this study.

	**Medical insurance claims**	**Long-term care insurance claims**	**Enrollment history**
Data period	April 2015 to March 2018		October 1991 to September 2018
Size	49991060 claims	5754329 claims	2934609 records
Providers	128 insurance providers	42 insurance providers	42 local governments

### Software

The anonymized medical and long-term care insurance claims were managed and processed on a specialized data platform [45]. The ANOVA-based filtering, the model training (i.e., GBDT, MLP, and SVM) and the calculation of the evaluation indicators (i.e., Precision, Recall, and F-measure) were implemented with Python 3.6.1 with NumPy 1.12.1 and Scikit-learn 0.18.1 [46].

## Results

Intensive analyses were conducted to validate the accuracy of the proposed prediction method.

### Comparison of multiclass and class-specific classification

First, we conducted a comparative study to compare two different modeling approaches: multiclass classification and class-specific classification. The designed prediction task was to predict future eligibility levels at 3 (June 2017), 6 (September 2017), and 12 (March 2018) months after previous insurance claims recorded from April 2016 to March 2017. Specifically, separate prediction models were designed for each approach, and the models were trained using the GBDT algorithm with insurance claims recorded from April 2016 to March 2017 and future eligibility levels recorded on June 2017, September 2017, and March 2018. We then evaluated the prediction accuracy of future eligibility levels of these three different timepoints [3 (June 2017), 6 (September 2017), and 12 (March 2018) months] after the previous insurance claims (April 2016 to March 2017). We used insurance claims from 137,101 individuals randomly selected from the entire individual set for the model training and then used insurance claims of another 137,101 individuals for the prediction validation (Table 3). For simplicity, we excluded individuals who withdrew from the regional public insurance during the training and prediction periods. Importantly, the training and test datasets were clearly separated to achieve fairness.

**Table 3:** Datasets for model training and prediction validation (for Tables 4–6).

	**Model training dataset**	**Prediction validation dataset**
Data period	April 2016 to March 2018	
Number of individuals	137101	137101
Selection	Randomly chosen	Remaining set

Table 4 summarizes the accuracy of the prediction models designed in the multiclass classification approach in predicting individuals’ future (at 3, 6, and 12 months after previous claims) eligibility levels of long-term care. For example, the RLTC5 group comprised 3,592 individuals on June 2017 (3 months after the previous claims), and the prediction model predicted the individuals in the RLTC5 group as: Precision, 0.922; Recall, 0.893; and F-measure, 0.907. Table 4 lists the evaluation indicators of each group for each prediction period (3, 6, and 12 months after previous claim) in the same manner. Table 4 also shows the (unweighted) average and weighted average as overall indicators. The prediction accuracy was highest for the 3-month prediction (weighted average F-measure = 0.949), followed by 6 and 12 months (weighted average F-measure = 0.924 and 0.873, respectively). In addition, estimations of 95% confidence intervals were also performed by resampling the test dataset 1,000 times by bootstrapping [39]. The prediction was highly confident, and the lower and upper limits of the 95% confidence intervals were all within ±0.001 for Precision, Recall, and F-measure for each group (data not shown). These results indicate that the models designed in the multiclass classification approach consistently offer a highly accurate prediction of individuals’ future eligibility (weighted average F-measure = 0.873 for 12-months prediction).

**Table 4:** Future eligibility level prediction based on multiclass classification and GBDT.

Prediction	3 months				6 months				12 months			

Evaluation indicator	P	R	F	N	P	R	F	N	P	R	F	N

RLCT5	0.922	0.893	0.907	3592	0.921	0.811	0.862	3972	0.921	0.668	0.775	4843
RLCT4	0.857	0.852	0.854	5086	0.824	0.761	0.791	5521	0.760	0.598	0.670	6487
RLCT3	0.852	0.838	0.845	6232	0.805	0.747	0.775	6548	0.707	0.606	0.653	7115
RLCT2	0.855	0.839	0.847	7849	0.806	0.758	0.781	8190	0.706	0.624	0.662	8744
RLCT1	0.864	0.832	0.848	7083	0.784	0.757	0.770	7174	0.622	0.631	0.627	7245
RS2	0.831	0.883	0.856	4986	0.786	0.814	0.800	5098	0.700	0.704	0.702	5245
RS1	0.848	0.782	0.814	3128	0.780	0.719	0.748	3112	0.681	0.621	0.649	3089
Ineligible	0.984	0.990	0.987	99145	0.969	0.990	0.980	97486	0.940	0.989	0.964	94333

AVG	0.877	0.863	0.870	–	0.834	0.795	0.813	–	0.755	0.680	0.713	–
WAVG	0.949	0.950	0.949	–	0.923	0.926	0.924	–	0.872	0.878	0.873	–

P, Precision; R, Recall; F, F-measure; AVG, unweighted average; WAVG, weighted average

**Fig. 2:**
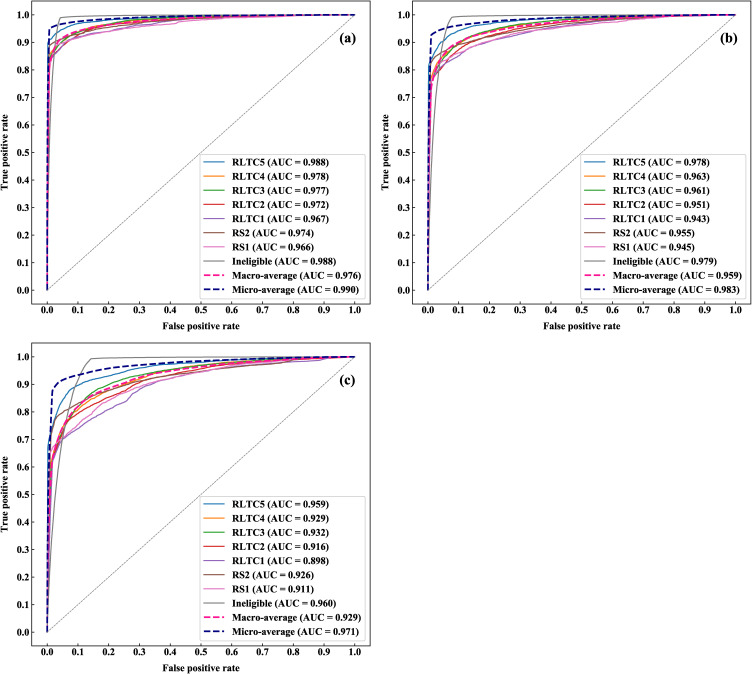
ROC curves and AUC values in the future eligibility level prediction. (a) 3-month prediction; (b) 6-month prediction; and (c) 12-month prediction.

Figure 2 presents receiver operating characteristic (ROC) curves and area under the ROC curve (AUC) values of the prediction models presented in Table 4. The (a) to (c) charts present the result of the 3, 6, and 12 months after prediction, respectively; a separate ROC curve is plotted and an AUC value is presented for each of the eligibility levels. In addition, these charts also show the macro and micro averages. The macro average treats each group equally, whereas the micro average treats each sample equally (i.e., weighting each group with its contribution). Specifically, the macro and micro averages correspond to the (unweighted) average and weighted average presented in Table 4. The charts indicate the multiclass classification approach offered a robustly highly accurate prediction of individuals’ future eligibility; the AUC value was the highest for the 3-month prediction (micro-averaged AUC = 0.990), followed by 6 and 12 months (micro-averaged AUC = 0.983 and 0.971, respectively).

**Fig. 3:**
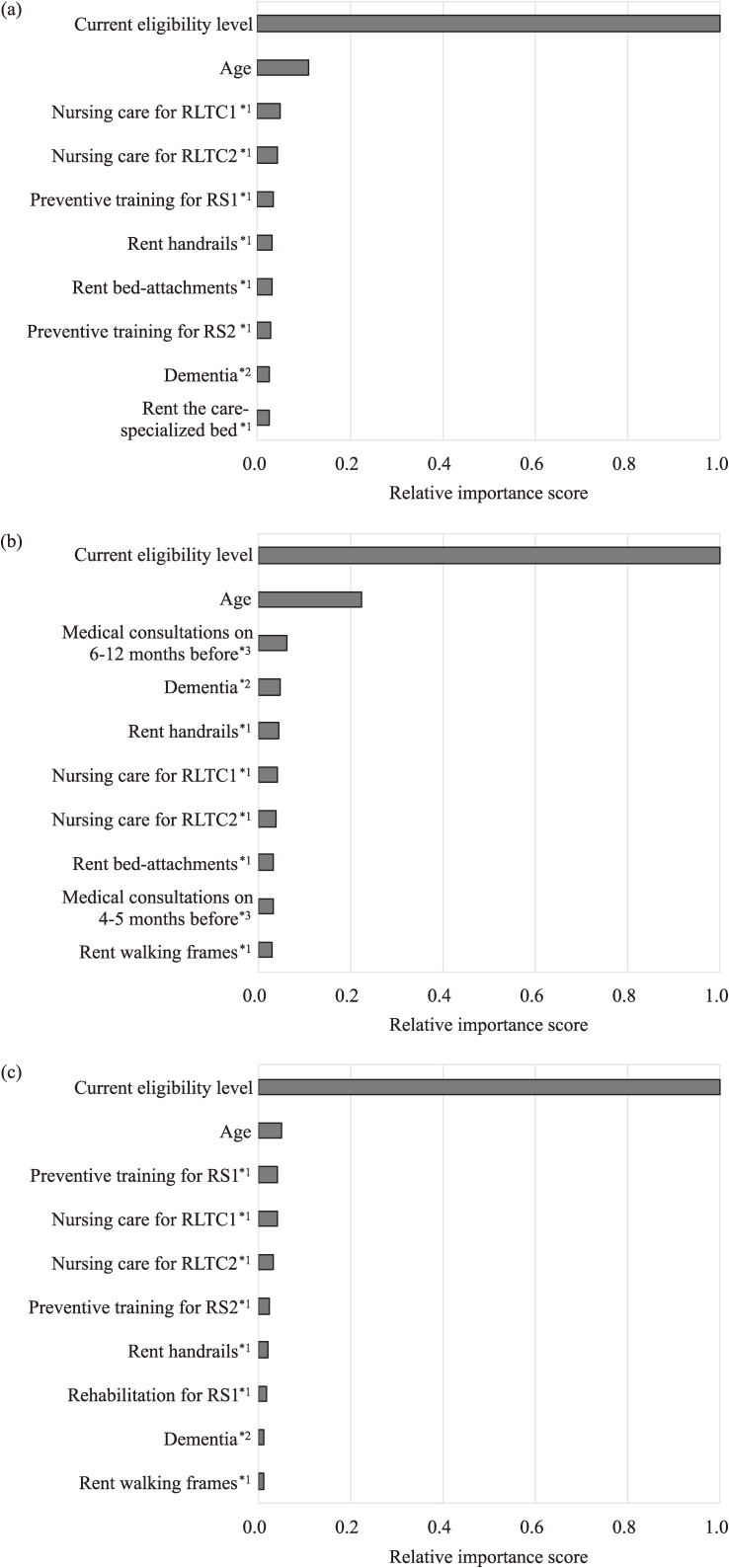
Top 10 feature variables of prediction models designed by multiclass classification and GBDT. (a) 3-month prediction; (b) 6-month prediction; and (c) 12-month prediction. 1*, frequency of occurrence of long-term care service code; 2*, frequency of occurrence of disease diagnosis code; and 3*, frequency of occurrence of medical treatment procedure code.

The important feature variables selected in the prediction models for predicting individuals’ eligibility levels at 3, 6, and 12-month prediction are shown in Fig. 3. The highly important features were roughly divided into three categories: (1) direct indicators of current health status, such as current eligibility levels, age, frequency of nursing care usage, and rent care-related equipment; (2) risk factors for worsened future healthcare status, such as dementia and frequency of past medical consultation; and (3) use of preventive care services to improve future healthcare status, such as training and rehabilitation.

Table 5 shows the prediction results provided by the class-specific classification approach, which also performed consistently well but yielded relatively lower accuracy (weighted average F-measure = 0.769 for 12-month prediction) compared with the multiclass classification approach. The 95% confidence interval values also showed a similar result. Originally, we expected that the imbalance of long-term care eligibility levels would decrease the prediction accuracy; thus, we used the class-specific classification approach to overcome the imbalance. However, the multiclass classification approach showed a higher prediction accuracy in practice.

**Table 5:** Future eligibility level prediction based on class-specific classification and GBDT.

Prediction	3 months				6 months				12 months			

Evaluation indicator	P	R	F	N	P	R	F	N	P	R	F	N

RLCT5	0.771	0.488	0.598	3592	0.682	0.414	0.515	3972	0.396	0.374	0.385	4843
RLCT4	0.627	0.816	0.709	5086	0.492	0.689	0.574	5521	0.305	0.312	0.308	6487
RLCT3	0.760	0.573	0.654	6232	0.643	0.591	0.616	6548	0.466	0.433	0.449	7115
RLCT2	0.618	0.813	0.702	7849	0.592	0.698	0.641	8190	0.457	0.517	0.485	8744
RLCT1	0.437	0.736	0.548	7083	0.280	0.573	0.377	7174	0.247	0.391	0.303	7245
RS2	0.680	0.582	0.628	4986	0.533	0.665	0.592	5098	0.516	0.678	0.586	5245
RS1	0.446	0.489	0.467	3128	0.485	0.466	0.475	3112	0.656	0.502	0.569	3089
Ineligible	0.961	0.907	0.933	99145	0.965	0.864	0.911	97486	0.953	0.896	0.924	94333

AVG	0.663	0.676	0.655	–	0.584	0.620	0.588	–	0.500	0.513	0.501	–
WAVG	0.866	0.842	0.849	–	0.837	0.789	0.806	–	0.785	0.758	0.769	–

P, Precision; R, Recall; F, F-measure; AVG, unweighted average; WAVG, weighted average

### Comparison of learning algorithms

A comparative study was conducted to compare the three different learning algorithms (GBDT, MLP, and SVM) (Table 6). We performed the same tests as those shown in Table 4 (the multiclass classification approach) with MLP and SVM, as well as GBDT. Comparative analysis indicated that, in practice, all learning algorithms yielded a high accuracy, and GBDT yielded slightly superior accuracy to the other two. The 95% confidence intervals revealed that the lower and upper limits were within ±0.001 for Precision, Recall, and F-measure for all cases.

**Table 6:** Future eligibility level prediction based on multiclass classification and three different learning algorithms.

Learning algorithm	GBDT			MLP			SVM		

Evaluation indicator (weighted average)	P	R	F	P	R	F	P	R	F

3-month prediction	0.949	0.950	0.949	0.947	0.947	0.947	0.947	0.948	0.947
6-month prediction	0.923	0.926	0.924	0.922	0.924	0.923	0.894	0.892	0.893
12-month prediction	0.872	0.878	0.873	0.869	0.875	0.870	0.825	0.829	0.823

GBDT, gradient-boosting decision tree; MLP, multilayer perceptron; SVM, support vector machine; P, Precision; R, Recall; F, F-measure.

### Validation test for different dataset periods with an identical time period for model training and validation

We further intensively evaluated the prediction method to verify its robustness for different time periods. We conducted the same tests as those presented in Table 4 using different dataset periods to evaluate the robustness of the developed prediction method. The original test constructed prediction models for 3, 6, and 12-month predictions from the previous dataset (April 2016 to March 2017) and evaluated the models with another test dataset for the same periods. We changed the dataset periods and performed the same test to confirm the robustness of the proposed prediction method.

Table 7 summarizes the evaluation using four different dataset periods: (1) April 2015 to March 2017; (2) July 2015 to June 2017; (3) October 2015 to September 2017; and (4) January 2016 to December 2017. Similar performance properties were observed for all dataset periods. Here, we have only presented the results for the multiclass classification and GBDT analysis, which exhibited superior performance in terms of practical accuracy, although the other cases showed similar results. The 95% confidence intervals indicated that the analyses were highly confident for each dataset period. These findings confirm that the proposed prediction method demonstrated practically high robustness for different dataset periods.

**Table 7:** Future eligibility level prediction using four different dataset periods.

Previous period	April 2015 to March 2016			July 2015 to June 2016			October 2015 to September 2016			January 2016 to December 2016		

Prediction period	April 2016 to March 2017			July 2016 to June 2017			October 2016 to September 2017			January 2017 to December 2017		

Evaluation indicator (weighted average)	P	R	F	P	R	F	P	R	F	P	R	F

3-month prediction	0.944	0.944	0.944	0.951	0.952	0.951	0.952	0.952	0.952	0.947	0.948	0.947
6-month prediction	0.917	0.920	0.918	0.923	0.925	0.924	0.921	0.923	0.921	0.916	0.918	0.916
12-month prediction	0.864	0.871	0.865	0.861	0.867	0.862	0.859	0.865	0.860	0.857	0.863	0.858

P, Precision; R, Recall; F, F-measure

### Validation test for different dataset periods with separate identical time periods for model training and validation

In the evaluation cases presented thus far, the prediction models were constructed from the training datasets of certain time periods, and the resulting prediction was evaluated using separate validation datasets for the same time periods. We separated the training and validation datasets to achieve fairness even though both were during the same time period. In contrast, the following analysis presents a scenario of “time-shift” prediction, where the training and validation were performed at different time periods with the same model.

We constructed prediction models to predict eligibility levels at 3, 6, and 12 months after previous insurance claims from April 2016 to March 2017 and applied the prediction models for the following time periods: (1) April 2015 to March 2016; (2) July 2015 to 2016; (3) October 2015 to September 2016; and (4) January to December 2016. We then investigated the prediction accuracy of each test case.

Table 8 summarizes the evaluation result. The time-shift prediction yielded slightly lower accuracy on average, while it demonstrated practically high accuracy (weighted average of F-measure = 0.857 for the 12-month prediction). Here, we have only presented the result obtained with GBDT as the other algorithms presented similar results. The lower and upper limits of the 95% confidence intervals were also within ±0.001 for each case. These findings confirm that the proposed prediction method performed robustly.

**Table 8:** Future eligibility level prediction using a training dataset period and four different validation dataset periods.

Previous period	April 2016 to March 2018											

Prediction period	April 2015 to March 2017			July 2015 to June 2017			October 2015 to September 2017			January 2016 to December 2017		

Evaluation indicator (weighted average)	P	R	F	P	R	F	P	R	F	P	R	F

3-month prediction	0.943	0.943	0.943	0.949	0.949	0.949	0.949	0.949	0.949	0.944	0.944	0.944
6-month prediction	0.915	0.917	0.916	0.919	0.921	0.920	0.917	0.919	0.917	0.912	0.914	0.912
12-month prediction	0.862	0.868	0.863	0.859	0.864	0.860	0.857	0.863	0.858	0.856	0.862	0.857

P, Precision; R, Recall; F, F-measure

## Discussion

The findings of the present study can be summarized as follows. (1) The prediction model based on multiclass classification and GBDT achieved high accuracy up to 12-month prediction (weighted average F-measure = 0.873). (2) Direct indicators of current health status (e.g., current eligibility levels and age), risk factors that worsen future healthcare status (e.g., dementia), and preventive care services to improve future healthcare status (e.g., training and rehabilitation) showed high importance in predicting future eligibility levels. (3) Contrary to our expectations, the multiclass classification approach consistently achieved significantly higher accuracy than the class-specific classification approach (weighted average F-measure = 0.873 and 0.769 for multiclass classification and class-specific classification, respectively). (4) GBDT, MLP, and SVM performed comparably, but GBDT consistently achieved slightly higher accuracy than the other algorithms (weighted average F-measure = 0.873, 0.870, and 0.823 for GBDT, MLP, and SVM, respectively). (5) Intensive validation tests indicated that the prediction method was highly robust (weighted average F-measure ≥ 0.857).

We also observed that the RS1 and RLTC1 groups showed lower prediction accuracy than the other groups. These findings require further investigation but may be because the individuals in the RS1 and RLTC1 groups had health conditions that were hard to distinguish from each other. Furthermore, the developed predictor occasionally failed to correctly predict sudden escalation of long-term care demand (likely due to deterioration of health status) and was particularly notable in long-range prediction cases. For example, in the case presented in Table 4, 30.6% of the RLTC1 individuals (12 months later or March 2018) were predicted to be any of the ineligible, RS1, or RS2 groups. We are currently working on employing focused learning techniques, such as the ordinal classification and recurrent neural network [RNN; e.g., gated recurrent unit (GRU) or long-term short memory network (LSTM)] [47–53], to improve the prediction accuracy, particularly of long-range prediction and in the RS1 and RLTC1 groups. In particular, the RNN-based models can deal with longitudinal dependencies between the input (feature variables) and output (future eligibility levels) information and improve the long-range prediction accuracy.

A major limitation of the present study is the sample size of the dataset. We used a 3-year insurance claims dataset provided by 170 regional public insurers in Gifu, Japan. Extending the datasets, such as expanding the data period and adding more insurers, would strengthen our prediction method. Scaling up the population size would enable the prediction models to perform significantly better, particularly for the RS1 and RLTC1 groups. Currently, there are >2,000 public insurers working in Japan [54], and there is significant room for improvement. Another major limitation of the study is that deceased patients were excluded from the training and validation datasets for simplicity. Some individuals may withdraw from or enroll in the insurance program and considering these individuals could improve future predictions. Grouping the deceased individuals into a “deceased” group could potentially resolve this problem, although further refinement of the models may be required.

On another front, the prediction model proposed in this study has been defined as a classification problem, in which a temporal variable (e.g., a month described in an insurance claim) is considered simply as a feature variable candidate but a temporal sequence property is not explicitly considered. Designing a time-series prediction model is an open problem; the time-series prediction model is useful for analyzing the temporal sequence property of future long-term care demands.

The prediction method proposed in this study was verified only with the dataset provided by the regional public insurers in Gifu as above noted. We expect that the method can be directly applied for other prefectural areas in Japan to predict their future long-term care demands. A comparative verification study with multi-prefectural datasets is ongoing. In addition, the basic framework for future prediction presented in this study is potentially applied and extended to a wide spectrum of prediction scenarios for estimating other future demands (e.g., medication, hospital admissions, and medical expenditure) from past insurance claim records. However, extensive work for model tuning and verification should be necessary for each specific prediction scenario. For example, GBDT consistently performed better than other algorithms in this study, but this observation does not automatically mean that GBDT works similar for other scenarios. In addition, extensive data preparation work might be demanded by other tasks; longer-term datasets could be necessary to predict the future conditions of slowly-progressing diseases.

Extending the future prediction to heterogeneous datasets is technically and socially interesting. For example, the association of lifestyle and psychosocial factors with a daily living competence is known [55]. The insurance claims do not describe those factors such as mental condition, daily habits, and social activities. Incorporating a dataset describing those factors to the prediction mechanism could further improve the demand prediction quality of long-term care.

Several previous studies have examined prediction of future service demand [1–11]. Wong et al. conducted a retrospective cohort study to determine the predictors (variables) of future long-term care of elderly patients using a multinomial logistic regression approach [56]. Worrall et al. proposed a method to forecast regional monthly long-term care demand and its cost using a time-series analysis approach, such as the ARIMA model, combining it with uncertainty parameters [57]. Xie et al. reported a method to predict patients’ hospitalization demand (i.e., the length of stay in hospital) using a decision tree algorithm from insurance claims [58]. Zhang et al. reported a comparison of machine learning algorithms for predicting activities of daily living status of elderly people 2–3 years in advance and reported that the C5.0 (decision tree) algorithm achieved the highest accuracy [59]. Cheng et al. proposed a framework for assessing chronic disease onset risk using sequential pattern mining techniques and reported that they could verify the sequential risk patterns on chronic obstructive pulmonary disease [60]. Mizuho reported a study to predict future long-term care demand from a previous enrollment dataset of long-term care eligibility [61]. Kasajima et al. reported a micro-simulation study to predict future economic cost of healthcare on the basis of national health surveys and existing cohort studies [62]. The present study is novel as we exploited information from medical and long-term insurance claims and used state-of-the-art learning algorithms to predict future long-term care demand at the individual level.

## Conclusion

The present study aimed to develop a novel and effective method to predict individual-level future long-term care demand using previous insurance claims data from medical and long-term care services. We designed discriminative models, which were then and trained and validated using medical and long-term care insurance claims and enrollment records provided by 170 regional public insurers covering 42 local government areas in Gifu, Japan. Overall analysis confirmed that the developed models offered a practically high accuracy for the prediction of individual-level future long-term care demand (Precision, 0.872–0.949; Recall, 0.878–0.950; and F-measure, 0.873–0.949). In the future, we will aim to resolve the remaining issues by extending the training and validation datasets and refining learning algorithms/modeling approaches to further improve the prediction method.

## Abbreviations

ANOVA: Analysis of variance
ARIMA: Autoregressive integrated moving average
AUC: Area under the ROC curve
AVG: Unweighted average
GBDT: Gradient-boosting decision tree
GRU: Gated recurrent network
LSTM: Long-term short memory
MLP: Multilayer perceptron
RLTC: Requiring long-term care
RNN: Recurrent neural network
ROC: Receiver operating characteristic
RS: Requiring support
SVM: Support vector machine
WAVG: Weighted average

## Declarations

### Availability of data and material

The Gifu dataset included in the study was provided by the regional public healthcare insurers in Gifu with the approval of the personal information protection commission in charge of each of the 42 local government areas and by contract with the local governments and the insurers. The dataset can be utilized for the limited use and its sharing with third parties is not allowed but needs the additional permission of the regional insurers in Gifu, Japan.
